# Effect of Potato Variety and Pretreatment Methods on Physicochemical and Sensory Properties of French Fries

**DOI:** 10.1002/fsn3.71188

**Published:** 2025-11-10

**Authors:** Tolina Kebede Regasa, Tilahun A. Teka, Addisalem Hailu Taye

**Affiliations:** ^1^ Department of Post‐Harvest Management, College of Agriculture and Veterinary Medicine Jimma University Jimma Ethiopia; ^2^ Department of Agro‐Food Processing Holeta Polytechnic College Holeta Ethiopia

**Keywords:** blanching, organoleptic properties, potato genotypes, potato processing, *Solanum tuberosum*

## Abstract

Improved potato varieties have been developed in Ethiopia with a focus on pre‐harvest quality traits; however, limited studies have evaluated their suitability for processing into value‐added French fries. In this study, oil uptake, physicochemical, and sensory properties of French fries prepared from six potato varieties (*Belete*, *Dagim*, *Bubu*, *Burka*, *Gudane*, and *Jalane*) were assessed following two pretreatment methods: soaking in 3% NaCl solution for 50 min and blanching in hot water at 85°C ± 2°C for 5 min, using a completely randomized design. Results revealed that French fries from *Belete* and *Burka* varieties treated with salt solution had the lowest oil uptake. The highest values were recorded for energy (487 Kcal), crude fat (33.6%), and crude fiber (3.99%) in untreated *Jalane*; crude protein in untreated *Burka* (3.93%) and *Gudane* (3.92%); crude ash (3.95%) and moisture content (26.7%) in soaked *Belete*; and utilizable carbohydrate in blanched *Belete* (51.2%). The lowest values were observed for energy (375 Kcal) and crude fat (19.4%) in soaked *Belete*; crude ash (1.69%) in blanched *Jalane*; moisture (17.8%) and utilizable carbohydrate (43.8%) in untreated *Jalane*; crude fiber (2.93%) in blanched *Jalane*; and crude protein (0.56%) in blanched *Dagim*. The highest mineral contents were found in untreated and soaked *Belete* and *Burka* varieties, with calcium (23.7 mg/100 g), iron (4.81 mg/100 g), and zinc (3.45 mg/100 g), while the lowest were observed in blanched *Gudane* for calcium (8.07 mg/100 g), and in blanched *Jalane* for both iron (1.60 mg/100 g) and zinc (1.83 mg/100 g). Sensory evaluation indicated that French fries prepared from treated *Belete* and *Burka* varieties were favorably rated by consumers in terms of color, crispness, and overall acceptability. These varieties are therefore recommended for large‐scale French fry production and potential commercialization.

## Graphical abstract

French fries prepared from six potato varieties (*Belete, Burka, Bubu, Gudane, Dagim,* and *Jalane*) were evaluated under blanching and salt‐soaking pretreatments. *Belete* and *Burka* varieties showed the lowest oil uptake and highest consumer acceptance in terms of crispness, color, and overall quality. Pretreatments significantly improved nutritional properties by reducing fat content and enhancing texture. These findings identify *Belete* and *Burka* as optimal varieties for French fry production and commercialization in Ethiopia. Effect of pretreatments on oil uptake (%) of French fries across potato varieties. *Belete, Burka, Bubu, Gudane, Dagim* and *Jalane* varieties.

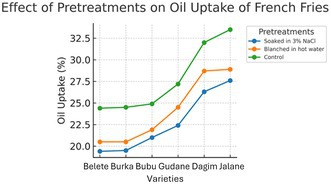

## 1 Introduction

Potatoes ( *Solanum tuberosum* L.) originated in the Andes mountains of South America (Reddy et al. 2018). It is a significant global food source, ranking third in terms of human consumption, and fourth in production after rice, wheat, and maize (Zhang et al. 2017; CIP 2017; FAOSTAT 2019). The economic significance of potatoes, particularly for farmers, has increased substantially, with over one billion people worldwide relying on potatoes as a staple food (Devaux et al. 2021).

Its adaptability to a wide range of agro‐climatic conditions, high yield potential, and nutritional value has driven its widespread cultivation and consumption across both developed and developing nations (Devaux et al. 2021). The potato sector provides significant employment opportunities throughout the value chain, from production and processing to distribution and food services, thereby contributing to poverty alleviation and rural development (Rob and Cattaneo 2021). In many countries, particularly in Asia, Africa, and Latin America, potato cultivation and processing have become viable income‐generating activities for smallholder farmers, youth, and women, reinforcing its significance as a tool for socio‐economic development (Adjei‐Nsiah et al. 2019). The increasing global demand for potatoes, driven by population growth and the expansion of the fast‐food and processed food industries, further highlights the crop's rising economic and commercial relevance worldwide.

Potatoes play a crucial role in ensuring food security and generating income for smallholder farmers in developing countries such as Ethiopia. There is an increasing demand for consumption of potatoes owing to their nutritional value and versatility in processing (WHO et al. 2020). However, the high water content of potatoes, comprising about 80% of the tuber, contributes to their perishability, leading to significant postharvest losses (Ngobese and Workneh 2018; Tajner‐Czopek et al. 2018). To mitigate this, the processing of potatoes into various stable products becomes crucial, offering a solution to reduce the postharvest losses of potatoes and enhance their value (Thapa and Thapa 2019).

Potatoes can be processed into a wide range of value‐added products, including French fries, flour, chips, flakes, starch granules, and other industrial derivatives (FAO 2008; Haverkort et al. 2023). These processing methods contribute to the production of wholesome, safe, and nutritious potato products, while simultaneously reducing postharvest losses and economically benefiting farmers for their produce (Haverkort et al. 2012; Thapa and Thapa 2019).

In particular, French fries are one of the most consumed snack products worldwide (Wijesinha‐Bettoni and Mouillé 2019). In Ethiopia, the consumption of French fries is rising due to urbanization, increased income levels in urban regions, and growing tourism (Haverkort et al. 2012). It has been reported that the quality of French fries depends on the potato variety and pretreatment used (Ndungutse 2019). Therefore, empirical evidence is essential for selecting suitable potato varieties based on their chemical compositions and consumer preferences.

The preparation of high‐quality French fries also involves pre‐treatment methods such as soaking in a salt solution and blanching (Pedro et al. 2006). Soaking in salt solution dehydrates potato slices through osmosis, resulting in crispier fries (Moyano and Berna 2002; Santis et al. 2007; Mosneaguta 2012). On the other hand, blanching is a critical step that inhibits enzymatic activity, particularly catalase, polyphenol oxidase, and peroxidase, which cause browning reactions. Furthermore, blanching enhances the color and texture of fries by gelatinizing surface starch, simultaneously reducing oil absorption and minimizing non‐enzymatic reactions caused by amino acids and reducing sugars (Liyanage et al. 2021; Punia Bangar et al. 2022).

In Ethiopia, potatoes are among the most important root and tuber crops widely cultivated and consumed across the nation, holding substantial potential to enhance food security (Tesfaye 2016; Worku 2019). With its high‐yielding capacity and suitability for diverse agro‐ecological zones, the potato surpasses major arable crops like wheat, rice, and maize in providing the highest energy yield per unit of land per day (Zaheer and Akhtar 2016). It is recognized as an affordable source of vital vitamins and minerals (Çalışkan et al. 2023).

Most previous research initiatives in Ethiopia on potatoes have focused on pre‐harvest activities such as production, yield improvement, agronomic performance, and disease resistance (Dersseh et al. 2016). There is a paucity of information about the suitability of improved potato varieties as affected by different pretreatment methods for the production of value‐added French fries. This research aims to investigate how different potato varieties and pretreatments (blanching and soaking) affect the nutritional value, physical characteristics, and consumer acceptance of French fries.

## 2 Materials and Methods

### 2.1 Experimental Materials

A total of 30 kg of six potato varieties (*Belete, Jalane, Burka, Gudane, Dagim*, and *Bubu*) was collected from Holeta Agricultural Research Center, Holeta, Ethiopia. The palm olein used for frying the French fries was purchased from the Geda supermarket in Jimma town. French fries production and subsequent quality evaluation were conducted in the laboratory of the Department of Postharvest Management, College of Agriculture and Veterinary Medicine at Jimma University.

### 2.2 Preliminary Works

**FIGURE 1:**
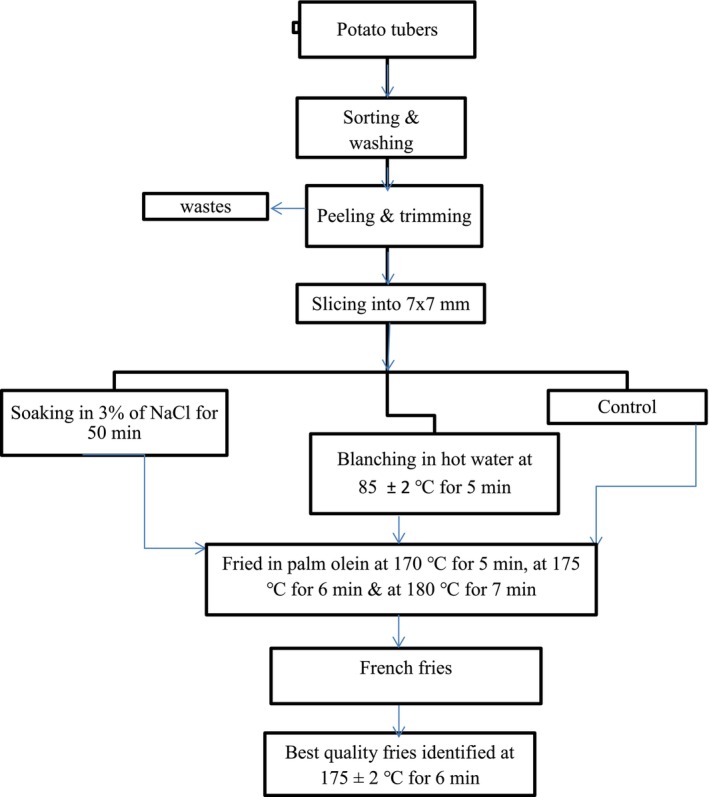
Flow diagram of preliminary works of the experiment.

In the preliminary work of the experiment, 15 selected potato tubers were washed, peeled, and sliced into 7 × 7 mm strips with a manual stainless steel potato cutter (French fries Cutter; Model No. MX‐002: England). Then, the strips were separately soaked in 3% NaCl for 50 min at a ratio of NaCl solution to potato strips of 8:1 (w/w) and blanched at a temperature of 85°C for 5 min (weight/water volume ratio of 1:20). After that, frying of slices was carried out at 170°C for 5 min, at 175°C for 6 min, and 180°C for 7 min in palm olein at a 200 g/2.5 L slices to oil ratio, until getting the fully fried slices, a crispy texture, and golden color according to recommendations by previous researchers (Zhang et al. 2017; Tajner‐Czopek et al. 2018; Das et al. 2021). Finally, based on the preliminary works of the study, a frying temperature of 175°C ± 2°C and a time of 6 min were selected for frying. Below, the flow diagram of the preliminary works has been indicated (Figure 1).

### 2.3 Experimental Setup and Design of Experiments

The experiment involved a completely randomized design with a 6*3 factorial arrangement, encompassing six potato varieties and two pretreatment methods (soaking in 3% sodium chloride solution for 50 min and blanching in hot water at 85°C ± 2°C for 5 min) and control samples. Along with a control, the experiment had 18 treatment combinations with three replications, which eventually resulted in 54 experimental units.

### 2.4 Frying Process

#### 2.4.1 Sample Preparation

From each of the six potato varieties, 15 uniform, damage‐free tubers were selected, washed, peeled, and cut into 7 × 7 mm strips using a manual French fry slicer (Wil van Loon 2012). Strips over 50 mm were divided for soaking in 3% sodium chloride for 50 min and for blanching in hot water at 85°C ± 2°C for 5 min, while the control batch was directly fried.

##### 2.4.1.1 Soaking

Soaking was carried out according to procedures reported by Bunger et al. (2003). The slices were soaked in a 3% NaCl solution at room temperature for 50 min at a ratio of NaCl solution to potato slices of 8:1 (w/w). After soaking, the samples were immediately drained on paper towels for 3 min. Then, the slices were ready to fry.

##### 2.4.1.2 Blanching

Samples were blanched according to Ngobese et al. (2017) and Kita et al. (2022). Strips were put in a stainless steel wire basket and blanched in hot distilled water at 85°C ± 2°C for 5 min with a constant slice weight to water volume ratio of 1:20 in the water bath (Model: Clifton NE 1–22/15136, England). Immediately after blanching, the slices were allowed to cool and drain on paper towels for 3 min.

##### 2.4.1.3 Frying

Next, samples were fried by using a double electric fryer (Model Number: UK‐ELF04; United Kingdom) in palm oil, at 175°C ± 2°C for 6 min. According to Alvarez et al. (2000), slices of 200 g/2.5 L of oil were used. After frying, samples for laboratory analysis were packed in high‐density polyethylene packages and placed in the refrigerator at 4°C. Fried samples for organoleptic analysis were immediately introduced to sensory analysis.

#### 2.4.2 Sample Preparation for Analysis

**FIGURE 2:**
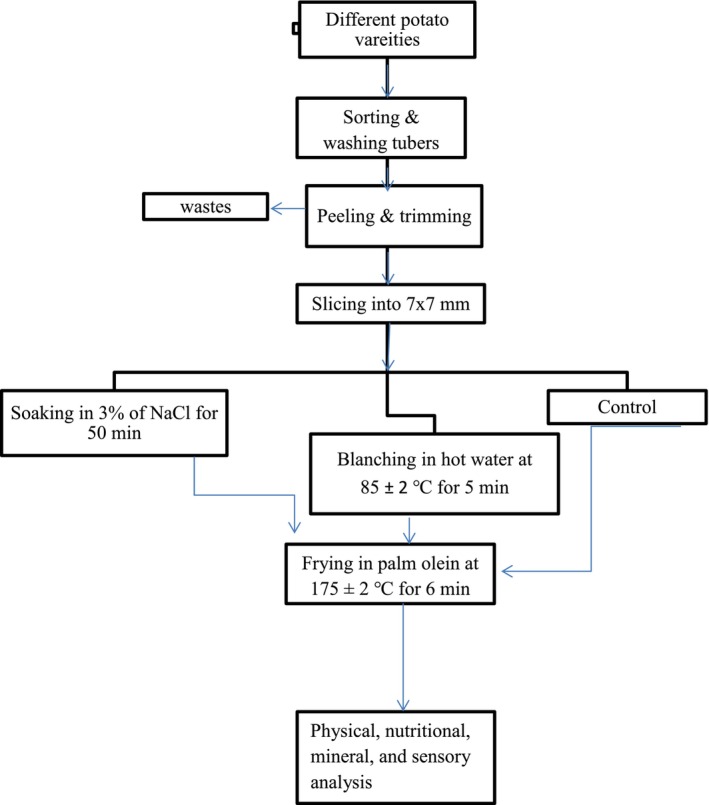
Flow diagram of the experimental framework of sample preparation, pretreating, frying, and analysis.

The French fries samples were prepared according to the procedures described by Motsara (2015) for objective analysis. First, the fries were dried in an oven (MODEL SX‐5‐12, China) at 55°C and then ground into powder by using a grinder (KARLBOLB D‐6072, Driech, West Germany). Then, they were sieved using a 0.25 mm sieve size. Finally, 100 g of the French fries powder was packed in high‐density polyethylene (HDPE) packing material for each treatment, and the analysis of the oil uptake, proximate compositions, and minerals was carried out. The general flow diagram of the experimental framework of sample preparation, pretreating of potato slices, frying, and analysis of various attributes has been indicated in Figure 2 below.

### 2.5 Determination of Initial Crude Fat Content and Oil Uptake

The initial crude fat content of the six varieties of fresh potatoes and fries was determined by the hexane solvent extraction method using Soxhlet equipment according to the AOAC (2000) approved method 960.39. Finally, the absorbed oil by the fries was calculated from the difference in the initial fat content of tubers (raw samples) and the products (French fries).

$$\text{Absorbed oils}=\text{product oils}\;\left(\text{fries}\right)-\text{sample oils}\;\left(\mathrm{raw}\;\text{potato}\right)$$

### 2.6 Determination of Physical Parameters

#### 2.6.1 Total Color

Color analysis was carried out using the HTWG Color Analyzer software. The samples were photographed under controlled circumstances in a special chamber with a black background. Three replications of the experiment were performed for both sample types (raw and fried). Then, using the software's color parameters (*L*, *a*, and *b*) total color changes were determined as per (Sobol et al. 2020; Dufera et al. 2022).

$$\Delta E=\sqrt{{\left(\Delta L\right)}^{2}+{\left(\Delta a\right)}^{2}+{\left(\Delta b\right)}^{2}}$$

where: Δ*E* = Total color change; Δ*L* = *L*−*L*_0_; Δ*α* = *α*−*α*_0_; Δ*b* = *b*−*b*_0_; *L* = Brightness of the fried sample; *L*_0_ = Brightness of the raw sample; *α* = Hue range of the colors red (+) and green (−) for fried sample; *α*_0_ = Hue range of the colors red (+) and green (−) for raw sample; *b* = Hue range of colors yellow (+) and blue (−) for fried sample; *b*_0_ = Hue range of colors yellow (+) and blue (−) for raw sample.

#### 2.6.2 Fries Hardness

The hardness measurements of the fries were determined by a texture analyzer according to the procedures (Tuta and Palazoğlu 2017). Texture analysis of French fries was conducted using the TA.XTplusC Texture Analyzer (Stable Micro Systems Ltd., Godalming, Surrey, UK), operated with Exponent Connect software. Experiments were conducted by placing the fries samples on the rig and driving the probe perpendicularly attached to the crosshead with a speed of 1 mm/s; the trigger force was 5 g and the travel distance of the probe was adjusted to 3 mm. Texture measurements were performed within 10 min after the potato fries were removed from the frying oil to prevent moisture absorption from the surrounding air and moisture loss from the inner fries to the crust, which would make the fries soggy and limp. Potato fries (soaked, blanched, and controlled) from each sample were analyzed for every three replications and the average data was recorded.

### 2.7 Determination of Proximate Composition

The proximate compositions of fries, such as moisture content, total ash, crude fiber, crude fat, and crude protein contents of fries, were determined by using AOAC (2000) methods of 925.09, 923.03, 962.09, 960.39, and 976.05, respectively. The utilizable carbohydrate was calculated by difference according to Oyelude et al. (2012).

### 2.8 Determination of Mineral Contents

Mineral analysis of calcium, iron, and zinc was performed using a 210 VGP Atomic Absorption Spectrophotometer (Buck Scientific, East Norwalk, CT, USA), equipped with a flame atomizer and operated with manufacturer‐supplied VGP software, following the American Association of Cereal Chemists (AACC 2000) Method 54‐21.

### 2.9 Sensory Evaluation

The organoleptic properties of the processed French fries were evaluated following the method described by Abong et al. (2009). Sensory evaluation was conducted in a controlled laboratory environment at the Department of Food Science and Technology, Jimma University, to ensure consistency in lighting, temperature, and sample presentation, following standard protocols. A total of 50 panelists, comprising 31 males and 19 females, were selected from the Department of Food Science and Post‐Harvest Technology, including both staff and students. Among them, 23 panelists were aged between 20 and 30 years, while 27 were between 31 and 40 years.

Each panelist received one coded sample at a time for evaluation, ensuring there was no bias due to sample order. The samples were freshly prepared and served in identical containers, labeled with random three‐digit codes. Panelists were also provided with a glass of water to cleanse their palate between samples. Each participant was given a score sheet to independently evaluate the samples based on color, crispness, flavor (taste + aroma), and overall acceptability. A 5‐point hedonic scale was used, where 1 = dislike extremely, 2 = dislike, 3 = neither like nor dislike, 4 = like, and 5 = like extremely. After the evaluation of all samples, the completed score sheets were collected for further analysis.

### 2.10 Subjects

The study protocol involving sensory evaluation was reviewed and approved by the Institutional Review Board (IRB) of Jimma University, College of Agriculture and Veterinary Medicine. Ethical clearance was granted prior to data collection, and informed consent was obtained from all participants before their involvement in the sensory evaluation.

### 2.11 Data Analysis

The data collected were analyzed using two‐way ANOVA of the Minitab statistical software version 19.1. Analyses were performed through analysis of variance (ANOVA) at 95% confidence and accepted at the level of significance *α* = 0.05, using Tukey's test to determine significant differences among means. Correlation between the oil uptake and proximate composition was conducted using Pearson's correlation method.

## 3 Results and Discussion

### 3.1 Physicochemical Properties

#### 3.1.1 Initial Crude Fat Content of Potato Varieties

The fat content of the six fresh cultivars ranged from 0.038% in the *Belete* variety to 0.300% in the *Jalane* variety (Table 1). Accordingly, the highest fat content was recorded in the *Bubu* variety (0.300%) while the lowest fat content (0.038%) was observed in *Belete*, closely followed by the *Burka* variety (0.066%). Variation in the crude fat content among the six potato varieties could be due to varietal differences similar to those reported by Gumul et al. (2011).

**TABLE 1:** Means of initial crude fat content and oil uptake of French fries.

Varieties	Initial crude fat (%)	Factors		Oil uptake (%)
		Pretreatments	Varieties	
*Belete*	0.038 ± 0.01^c^	Soaked in 3% of NaCl concentration for 50 min	*Belete*	19.4 ± 0.17^i^
*Burka*	0.066 ± 0.02^c^		*Burka*	19.5 ± 0.21^i^
*Bubu*	0.300 ± 0.03^a^		*Bubu*	21.0 ± 0.15^h^
*Gudane*	0.115 ± 0.04^bc^		*Gudane*	22.4 ± 0.20^g^
*Dagim*	0.193 ± 0.04^ab^		*Dagim*	26.3 ± 0.07^e^
*Jalane*	0.084 ± 0.01^bc^		*Jalane*	27.6 ± 0.14^d^
		Blanched in hot water at 85°C ± 2°C for 5 min	*Belete*	20.5 ± 0.18^h^
			*Burka*	20.5 ± 0.17^h^
			*Bubu*	21.9 ± 0.12^g^
			*Gudane*	24.5 ± 0.17^f^
			*Dagim*	28.7 ± 0.06^c^
			*Jalane*	28.9 ± 0.04^c^
		Untreated sample (control)	*Belete*	24.4 ± 0.17^f^
			*Burka*	24.5 ± 0.10^f^
			*Bubu*	24.9 ± 0.10^f^
			*Gudane*	27.2 ± 0.20^d^
			*Dagim*	32.0 ± 0.09^b^
			*Jalane*	33.5 ± 0.23^a^
CV	0.23			0.98

*Note:* Results are mean values of triplicate determination (reading) ± standard error. Means with the different superscript letters down the column are significantly different (*p* < 0.05).

Abbreviation: CV, coefficient of variation.

#### 3.1.2 Oil Uptake Capacity of French Fries

According to Table 1, the mean oil uptake ranged from 19.4% for fries of the *Belete* variety soaked in 3% NaCl solution to 33.5% for the control sample of the *Jalane* variety. The lowest oil uptake (19.4% and 19.5%) was observed in French fries of the *Belete* and *Burka* varieties soaked in NaCl solution before frying. In contrast, the highest oil uptake (33.5%) was recorded in untreated French fries of the *Jalane* variety, followed by the *Dagim* variety (32.0%). The high oil uptake in the untreated *Jalane* fries could be attributed to the absence of pretreatment and varietal differences, which may have increased surface porosity and oil absorption during frying.

In other hand, potatoes with higher dry matter content were reported to lose less water and absorb less oil during frying due to their compact internal cell structure (Ndungutse 2019). The lowest oil uptake could also be attributed to soaking potato slices in a salt solution, which can harden the surface of the slices and reduce the oil uptake of the French fries. Likewise, soaking slices in salt solution before frying has decreased the oil absorption capacity of the fries by creating a surface barrier through the osmosis process, sealing intercellular pores by dehydration, and reducing the final oil content of fries (Bunger et al. 2003).

The highest oil uptake of the French fries might be because of varietal differences and the absence of pretreating of potato slices. In agreement with this, untreated (control) potato slices were found to absorb more oil than pretreated strips during frying (Kizito et al. 2015). Based on the oil content of the French fries, potato varieties could be listed in their descending order as follows: *Jalane*>*Dagim*>*Gudane*>*Bubu*>*Burka*>*Belete*.

### 3.2 Physical Characteristics of French Fries

#### 3.2.1 Total Color Change of French Fries

**FIGURE 3:**
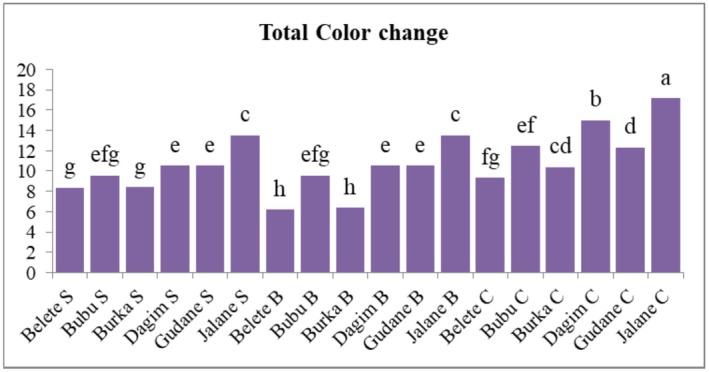
The effects of variety and pretreatment on the total color change of French fries. Data represent the mean ± standard error of triplicate determinations (*n* = 3). Means with different superscript letters indicate statistically significant differences among treatments at the 95% confidence level (*p* < 0.05). a–h letters = mean separation for varieties and pretreatments; B, blanche; C, control and S, soaked.

As indicated in Figure 3, the mean values of color change ranged from 6.19 in the blanched *Belete* to 17.2 in the control sample of the *Jalane* variety. The highest total color change was recorded for French fries of untreated potatoes. The highest total color change in French fries of untreated potatoes might be due to the removal of a high proportion of moisture during frying, varietal differences, and enzymatic and non‐enzymatic reactions, or a reaction of amino acids with reducing sugars in potatoes, in agreement with previous reports (van Loon 2012; Vinci et al. 2012).

The lowest color change in the French fries might be due to pretreatments applied, and cultivars. Pretreatments of sliced potatoes before frying were observed to produce a golden or yellowish color of French fries with acceptable color in a previous study (Mosneaguta 2012). In support of this, Xiao et al. (2017) reported that blanching of slices before frying enhanced the appearance of potato French fries. This is caused by inactivating browning reactions causing enzymes (catalases), leaching reducing sugar and amino acids content which would cause a Maillard reaction, and result in an undesirable dark color to the French fries.

#### 3.2.2 French Fries Hardness

**FIGURE 4:**
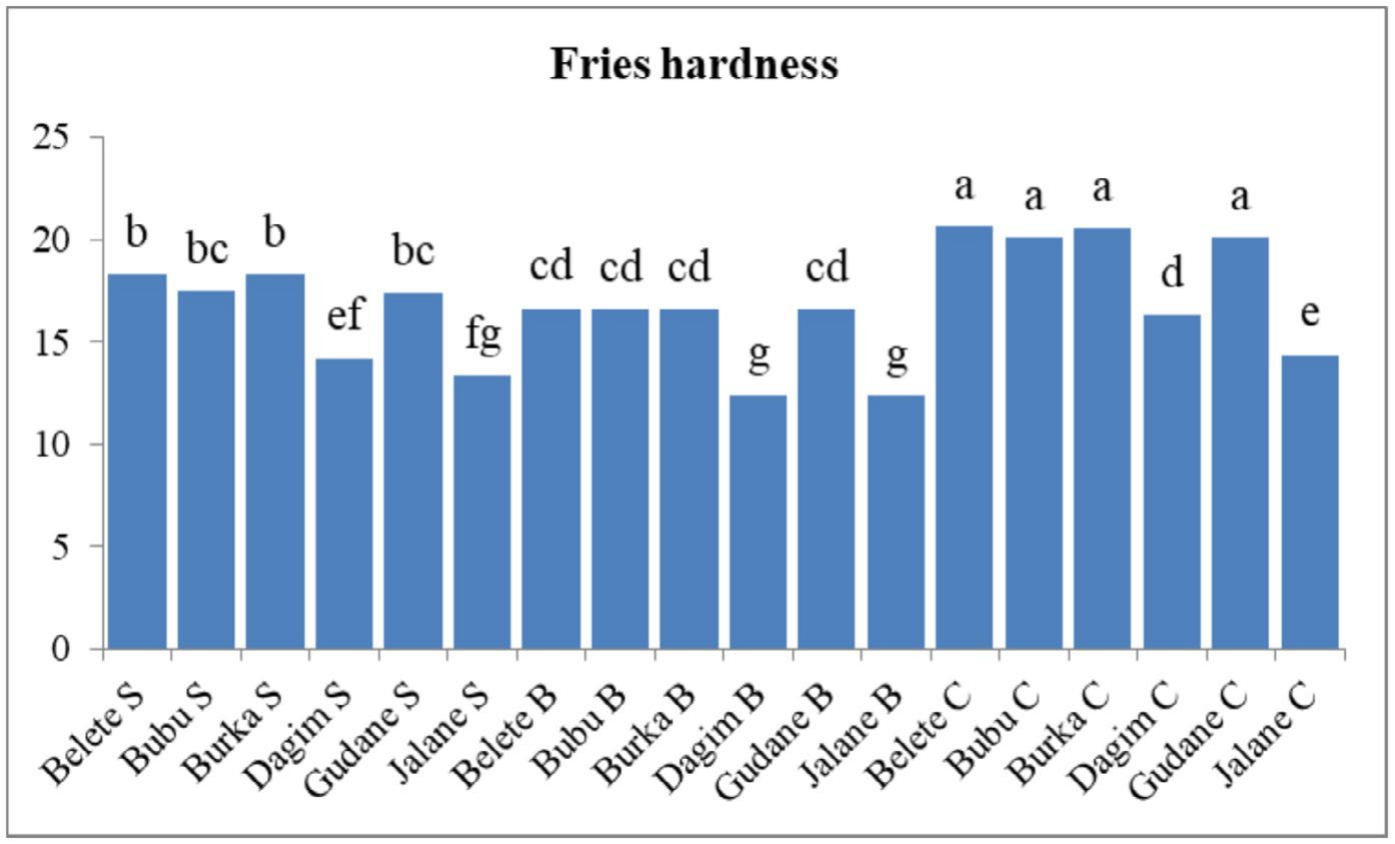
The effects of variety and pretreatment on the hardness of French fries. Data represent the mean ± standard error of triplicate determinations (*n* = 3). Means with different superscript letters indicate statistically significant differences among treatments at the 95% confidence level (*p* < 0.05). a–g letters = mean separation for varieties and pretreatments; B, blanche; C, control and S, soaked.

The mean hardness of potato fries ranged from 12.3 to 20.6 N, with the lowest value observed in the *Jalane* variety blanched in hot water at 85°C and the highest in the *Belete* variety (control). The highest hardness value was observed in untreated fries of *Belete*, *Burka* and *Gudane* potato varieties (20.6 N and 20.1 N). The highest hardness value observed in the control products may be attributed to the high dry matter content of the potato variety, denser microstructure at the surface, presence of hard cells, slightly greater water loss during frying, and the absence of pretreatment before frying. Variations in the hardness of French fries may result from varietal differences among the potatoes and the pretreatments applied, consistent with findings reported by Graham‐Acquaah et al. (2015). The variation in hardness among potato varieties and pretreatments is clearly illustrated in Figure 4.

The lowest value recorded may be attributed to the physical improvements of potato slices resulting from hot water blanching. This finding is supported by Su et al. (2022), who reported that blanching prior to frying can enhance the texture of the product by reducing surface structural hardness (i.e., decreasing density), allowing the formation of oil pockets or air cells, and thereby lowering the peak force required during texture analysis.

### 3.3 Proximate Composition

#### 3.3.1 Moisture Contents

As presented in Table 2, the average moisture content of French fries ranged from 17.8% in the untreated *Jalane* variety to 26.7% in the *Belete* variety pre‐soaked in a 3% NaCl solution prior to frying. The highest moisture contents were recorded in the *Belete* (26.7%) and *Burka* (26.6%) varieties treated with NaCl solution. This may be attributed to the interaction between varietal cellular composition (i.e., highly structured cells) and osmotic effects induced by the salt solution, which may have limited moisture loss during frying. Consistent with this finding, Pedreschi (2012) reported that potatoes with high dry matter content tend to retain more moisture during frying due to their densely structured internal cells. On the other hand, the lowest moisture contents were observed in the untreated fries of Jalane (17.8%) and Dagim (18.1%) varieties, likely due to greater moisture evaporation during frying. This observation aligns with the findings of Setiady et al. (2009), who noted that lower moisture content in fries is associated with lower varietal dry matter and increased microstructural porosity, which facilitates rapid moisture loss during frying and promotes greater oil absorption.

**TABLE 2:** Effects of variety and pretreatments on proximate compositions of French fries.

Factors		Proximate compositions in dry base						
Pretreatments	Varieties	MC%	CAC%	CFbC%	CFC%	CPC%	UCC%	Energy (Kcal)
Soaked in 3% of NaCl concentration for 50 min	*Belete*	26.7 ± 0.18^a^	3.95 ± 0.02^a^	3.41 ± 0.01^c^	19.4 ± 0.04^i^	3.57 ± 0.04^a^	46.4 ± 0.18^ef^	375 ± 1.55 ^i^
	*Burka*	26.6 ± 0.20^a^	3.94 ± 0.02^a^	3.43 ± 0.02^c^	19.6 ± 0.21^i^	3.92 ± 0.25^a^	45.9 ± 0.19^fgh^	376 ± 1.73^i^
	*Bubu*	25.5 ± 0.24^b^	3.63 ± 0.02^bc^	3.58 ± 0.04^bc^	21.3 ± 0.15^h^	2.89 ± 0.05^b^	46.6 ± 0.14^ef^	390 ± 1.69^h^
	*Gudane*	23.4 ± 0.14^d^	3.40 ± 0.03^cd^	3.65 ± 0.07^bc^	22.5 ± 0.20^g^	3.89 ± 0.06^a^	46.8 ± 0.27^ef^	405 ± 0.51^g^
	*Dagim*	22.7 ± 0.20^de^	2.96 ± 0.04^ef^	3.80 ± 0.03^ab^	26.5 ± 0.07^e^	1.89 ± 0.05^d^	45.9 ± 0.21^efgh^	430 ± 1.07^e^
	*Jalane*	22.3 ± 0.21^ef^	2.76 ± 0.09^fg^	3.98 ± 0.01^a^	27.7 ± 0.14^d^	2.38 ± 0.05^c^	44.9 ± 0.12^hi^	438 ± 1.20^d^
Blanched in hot water at 85°C ± 2°C for 5 min	*Belete*	24.7 ± 0.11^bc^	2.37 ± 0.07^hi^	2.45 ± 0.03^e^	20.5 ± 0.18^h^	1.19 ± 0.06^e^	51.2 ± 0.28^a^	395 ± 1.41^h^
	*Burka*	24.7 ± 0.13^bc^	2.36 ± 0.07^hi^	2.48 ± 0.04^e^	20.6 ± 0.12^h^	2.03 ± 0.06^cd^	50.4 ± 0.09^ab^	395 ± 1.59^h^
	*Bubu*	24.4 ± 0.11^c^	2.19 ± 0.08^ij^	2.60 ± 0.04^e^	22.2 ± 0.20^g^	1.06 ± 0.04^e^	50.1 ± 0.05^b^	404 ± 1.30^g^
	*Gudane*	23.4 ± 0.21^d^	2.03 ± 0.09^jk^	2.66 ± 0.06^de^	24.6 ± 0.17^f^	2.00 ± 0.06^cd^	48.0 ± 0.20^c^	421 ± 0.86^f^
	*Dagim*	21.8 ± 0.17^ef^	1.76 ± 0.06^kl^	2.69 ± 0.17^de^	28.9 ± 0.06^c^	0.557 ± 0.04^f^	47.0 ± 0.34^cde^	450 ± 1.04 ^c^
	*Jalane*	21.6 ± 0.12^fg^	1.69 ± 0.07 ^l^	2.93 ± 0.04^d^	28.9 ± 0.04^c^	0.923 ± 0.04^ef^	46.9 ± 0.21^def^	452 ± 0.50^c^
Untreated sample (control)	*Belete*	21.5 ± 0.28^fg^	3.73 ± 0.02^ab^	3.43 ± 0.01^c^	24.5 ± 0.17^f^	3.56 ± 0.04^a^	46.7 ± 0.39^ef^	421 ± 0.99^f^
	*Burka*	21.5 ± 0.26^fgh^	3.72 ± 0.02^ab^	3.44 ± 0.02^c^	24.6 ± 0.10^f^	3.93 ± 0.03^a^	46.2 ± 0.28^efg^	422 ± 1.12^f^
	*Bubu*	20.6 ± 0.23^gh^	3.40 ± 0.01^cd^	3.60 ± 0.05^bc^	25.2 ± 0.10^f^	2.90 ± 0.05^b^	47.9 ± 0.14^cd^	430 ± 1.43^e^
	*Gudane*	20.5 ± 0.25 h	3.23 ± 0.06^de^	3.67 ± 0.07^bc^	27.3 ± 0.20^de^	3.92 ± 0.06^a^	45.1 ± 0.12 ^h^	442 ± 1.95^d^
	*Dagim*	18.1 ± 0.09 ^i^	2.60 ± 0.02^gh^	3.82 ± 0.03^ab^	32.2 ± 0.09^b^	1.90 ± 0.05^d^	45.2 ± 0.06^gh^	478 ± 0.38^b^
	*Jalane*	17.8 ± 0.15^i^	2.49 ± 0.01^h^	3.99 ± 0.01^a^	33.6 ± 0.23^a^	2.39 ± 0.05^c^	43.8 ± 0.087^i^	487 ± 1.80^a^
CV		1.48	2.57	2.44	0.98	5.29	0.32	0.52

*Note:* Results are mean values of triplicate determination (reading) ± standard error. The mean with the different letters across the column is significantly different (*p* < 0.05).

Abbreviations: CAC, crude ash content; CFbC, crude fiber content; CFC, crude fat content; CPC, crude protein content; CV, coefficient of variation; MC, moisture content; UCC, utilizable carbohydrate content.

#### 3.3.2 Crude Ash Contents

As indicated in Table 2, the ash content of French fries ranged from 1.61% in the *Jalane* variety blanched in hot water at 85°C to 3.95% in the *Belete* variety soaked in a 3% NaCl solution prior to frying. The higher ash contents observed in the *Belete* (3.95%) and *Burka* (3.94%) varieties soaked in NaCl solution may be attributed to the absorption of minerals from the salt solution, as well as inherent varietal differences. This is supported by the findings of Arum et al. (2022), who reported that soaking potato slices in NaCl solution prior to frying increases the ash content of French fries. Conversely, the lowest ash content (1.61%) recorded in the blanched *Jalane* variety may be due to the leaching of minerals from the potato slices into the blanching water. Similarly, Ngobese et al. (2017), reported that blanching potato slices in water reduces the ash content after frying, likely due to mineral loss during the blanching process.

#### 3.3.3 Crude Fiber Contents

According to Table 2, the fiber content of the French fries ranged from 2.45% in the *Belete* variety blanched in hot water at 85°C to 3.99% in the untreated *Jalane* variety. The highest crude fiber contents of 3.99% and 3.98% were recorded in French fries of the *Jalane* variety, untreated and soaked in 3% NaCl concentration, respectively. This might be due to varietal differences, the frying process, and higher moisture removed in French fries. The findings from previous studies showed that frying potato slices makes fibers more concentrated and increases the contents in French fries compared to raw or boiled potatoes (Pedreschi 2012; Asokapandian et al. 2019). The lowest crude fiber content of the French fries made from potato slices that were subjected to blanching before frying, could be a potential reason to assume that these losses were due to the leaching of water‐soluble fibers from the slices into the blanching water (Marzuki et al. 2021).

#### 3.3.4 Crude Fat Contents

As shown in Table 2, the crude fat content of French fries is influenced by both the potato variety and the absorption of cooking oil during frying. The mean fat content ranged from 19.4% in the *Belete* variety soaked in a 3% NaCl solution to 33.6% in the untreated (control) *Jalane* variety. The highest fat content (33.6% and 32.2%) was observed in French fries of untreated *Jalane* and *Dagim* varieties. The lowest crude fat content measured in French fries of *Belete* and *Burka* varieties might be related to low moisture loss during frying and salt concentration effects. This observation agrees with previous studies in which potato slices that lost low moisture during frying absorb less oil by the French fries (Pedreschi 2012). The salt concentration can decrease the oil uptake of potato slices during frying by reducing surface moisture content through the osmosis process and sealing intercellular pores by dehydration according to previous reports (Bunger et al. 2003; Santis et al. 2007).

The elevated crude fat content observed in this study is likely attributable to substantial moisture loss during frying. It was reported that untreated potato slices absorb more oil during frying due to microstructural changes (porosity) and more oil replaces the evaporated water (Pedreschi 2012). In this study, the crude fat contents range was in agreement with the results reported in previous studies where the fat content in industrially prepared French fries is up to 36% while domestically fried could be increased to 40%–50% (Stastny et al. 2014; Kizito et al. 2015; Ngobese et al. 2017).

#### 3.3.5 Crude Protein Contents

As presented in Table 2, the mean protein content of the French fries ranged from 0.557% in the *Dagim* variety blanched in hot water at 85°C to 3.93% in the untreated (control) *Burka* variety. Higher protein contents (3.92%, 3.89%, and 3.57%) recorded in fries of *Burka*, *Gudane*, and *Belete* varieties, respectively treated with a 3% salt solution might be due to varietal differences and the insignificant effect of NaCl concentration on potato protein content. The high protein content of potato French fries is within the range (3.5%–7.27%) reported by Ngobese et al. (2017) and Jaggan et al. (2020).

The lowest protein content recorded in fried potato varieties subjected to blanching at (85°C) before frying could be attributed to the higher degree of leaching (loss) of water‐soluble protein components in blanching water. The decrease in protein content could be due to the leaching of soluble protein components (losses of water‐soluble amino acids) from the potato slices to water during blanching due to the degree of change in the microstructure (Ngobese and Workneh 2018). According to Abong et al. (2009) and Ooko (2008), the amount of French fries protein reported had an average of 2.5% and it is comparable with most other roots and tubers, except for cassava, which has only half of this amount. As compared to other crops like cereals, the biological value of potato protein is high and contains lysine amino acid which is not available in other cereals (Abong et al. 2009).

#### 3.3.6 Utilizable Carbohydrate Contents

As indicated in Table 2, the carbohydrate content of the French fries ranged from 43.8% in the untreated (control) *Jalane* variety to 51.2% in the *Belete* variety blanched in hot water at 85°C. The French fries prepared from *Belete* varieties blanched in water at 85°C had the highest carbohydrate contents (51.2%). French fries of the untreated *Jalane* variety had the lowest carbohydrate content (43.8%). The increases in carbohydrate contents of blanched French fries might be due to low values obtained for other parameters in blanched fries. In French fries, carbohydrates account for the bulk of the fries and serve as a good energy source (Zaheer and Akhtar 2016). Since the results of carbohydrate contents were calculated by difference, a decrease in fiber, protein, ash, and moisture contents of the fried French fries will ultimately affect the value of carbohydrate contents and contribute to the observed increases. The results of the carbohydrate contents of the French fries were in agreement with the findings reported by different researchers on French fries (Ooko 2008; Jaggan et al. 2020).

#### 3.3.7 Gross Energy

The main contributors to the calorific value of French fries are carbohydrates—the primary component of potatoes—and the oil absorbed during frying. The Table 2 shows, the energy content of the fries ranged from 375 kcal/100 g in the *Belete* variety soaked in NaCl solution to 487 kcal/100 g in the untreated (control) *Jalane* variety. The calorific value was highest in French fries of the untreated *Jalane* variety (487 kcal/100 g). The lowest calorific value content of French fries (375 kcal/100 g and 376 kcal/100 g) was observed in *Belete* and *Burka* cultivars treated with 3% NaCl concentration. The results indicated that the energy value of untreated fried products was undoubtedly increased, and this might be due to the high amount of oils absorbed during frying. The lowest calorific values might be due to lower fat absorbed at the time of the frying process because of the varietal differences and pretreatment of strips applied before frying. The main contributor to the calorific value of French fries is carbohydrates, which are the major component of potatoes, and the absorbed oil by slices during frying.

### 3.4 Mineral Contents of French Fries

As explained in Table 3, the mean calcium content of the French fries ranged from 8.07 mg/100 g in the *Gudane* variety blanched at 85°C to 23.7 mg/100 g in the untreated *Belete* variety. The high calcium content (23.7 mg/100 g and 23.6mg/100 g) measured in French fries of the untreated and soaked *Belete* variety in sodium chloride solution might be due to varieties and insignificant changes in soaking strips in salt before frying (Ngobese and Workneh 2018). The decrease in the calcium contents of French fries could be explained by the leaching of calcium from strips to water during blanching (Ngobese and Workneh 2018). The lowest calcium contents (1.60 mg/100 g and 1.96 mg/100 g) were registered in French fries of the *Jalane* and *Dagim* varieties subjected to blanching.

**TABLE 3:** The effects of variety and pretreatments on the mineral contents of French fries.

Factors		Minerals		
Pretreatments	Varieties	Calcium (mg/100 g)	Iron (mg/100 g)	Zinc (mg/100 g)
Soaked in 3% of NaCl concentration for 50 min	*Belete*	23.6 ± 0.17^a^	4.56 ± 0.01^a^	3.44 ± 0.17^a^
	*Burka*	21.3 ± 0.23^b^	4.80 ± 0.01^a^	3.37 ± 0.07^a^
	*Bubu*	18.5 ± 0.27^cd^	3.51 ± 0.01^b^	2.62 ± 0.06^b^
	*Jalane*	17.7 ± 0.10^d^	2.00 ± 0.02^c^	2.03 ± 0.04^de^
	*Gudane*	11.5 ± 0.25^g^	3.38 ± 0.01^b^	2.38 ± 0.01^bc^
	*Dagim*	14.6 ± 0.10^e^	2.05 ± 0.02^c^	2.22 ± 0.03^cd^
Blanched in hot water at 85°C ± 2°C for 5 min	*Belete*	19.2 ± 0.19^c^	3.27 ± 0.04^b^	2.31 ± 0.09^cd^
	*Burka*	18.6 ± 0.03^cd^	3.53 ± 0.01^b^	2.30 ± 0.06^cd^
	*Bubu*	13.5 ± 0.29^f^	2.30 ± 0.01^c^	2.25 ± 0.04^cd^
	*Jalane*	13.3 ± 0.10^f^	1.60 ± 0.30^cd^	1.83 ± 0.09^e^
	*Gudane*	8.07 ± 0.09^i^	2.20 ± 0.01^c^	2.14 ± 0.05^cd^
	*Dagim*	9.41 ± 0.30^h^	1.96 ± 0.04^cd^	2.05 ± 0.06^de^
Untreated sample (Control)	*Belete*	23.7 ± 0.19^a^	4.59 ± 0.01^a^	3.45 ± 0.01^a^
	*Burka*	21.4 ± 0.23^b^	4.81 ± 0.01^a^	3.37 ± 0.04^a^
	*Bubu*	18.6 ± 0.27^cd^	3.58 ± 0.02^b^	2.63 ± 0.07^b^
	*Jalane*	17.7 ± 0.21^d^	2.02 ± 0.01^c^	2.06 ± 0.08^de^
	*Gudane*	11.5 ± 0.23^g^	3.39 ± 0.01^b^	2.42 ± 0.02^bc^
	*Dagim*	14.6 ± 0.13^e^	2.06 ± 0.02^c^	2.26 ± 0.06^cd^
CV		2.17	3.98	3.79

*Note:* Results are mean values of triplicate determination (reading) ± standard error. The mean with the different letters across the column is significantly different (*p* < 0.05).

Abbreviation: CV, coefficient of variation.

As presented in Table 3, the mean iron content of French fries ranged from 1.60 mg/100 g in the *Jalane* variety blanched in hot water at 85°C to 4.81 mg/100 g in the untreated *Burka* variety. The highest iron contents of French fries of untreated *Burka* and *Belete*, soaked *Burka* and *Belete* varieties were 4.81 mg/100 g, 4.80 mg/100 g, 4.59 mg/100 g, and 4.56 mg/100 g, respectively. These results are comparable with values (2.65 mg/100 g—4.54 mg/100 g) reported by Abong et al. (2009) and slightly lower than the reported (9.37 mg/100 g–15.79 mg/100 g) by Jaggan et al. (2020). A decrement in iron content was observed for French fries that were blanched, and this might be due to the leaching of iron into blanching water (Barrett et al. 2010).

As presented in Table 3, the mean zinc content of French fries ranged from 1.83 mg/100 g in the *Jalane* variety blanched in hot water at 85°C to 3.45 mg/100 g in the untreated *Belete* variety. Zinc contents (3.45 mg/100 g, 3.44 mg/100 g, 3.37 mg/100 g, and 3.37 mg/100 g) were found in higher amounts in French fries of untreated *Belete* soaked in 3% NaCl, untreated *Burka*, and *Burka* soaked in 3% NaCl, respectively. The higher zinc content observed may be attributed to agricultural practices, ecological conditions, and the pretreatments applied—particularly the pre‐soaking of slices in sodium chloride solution—which may have contributed to minimal changes in zinc content.

The lowest zinc content (1.83 mg/100 g) was measured in French fries of the *Jalane* variety whose slices were blanched in hot water at 85°C. This result indicates that blanching significantly reduced the zinc content of the fries, likely due to the leaching of zinc from the potato slices into the blanching water. This explanation is supported by previous studies, which reported similar losses of zinc during blanching (Abong et al. 2009; Ngobese and Workneh 2018).

### 3.5 Sensory Properties

#### 3.5.1 Color

The color rating scores of French fries ranged from 1.78 (disliked) for untreated *Jalane* products to 5.00 (extremely liked) for the *Burka* variety blanched at 85°C and soaked in 3% NaCl (Table 4). Panelists rated the yellow‐colored *Burka* variety the highest (5.00/extremely liked), likely due to its naturally appealing color and effective pretreatment, which is supported by similar findings in studies by Zhang et al. (2017) and Furrer et al. (2017), where consumers preferred golden‐yellow French fries made from yellow‐flesh potato varieties. Kizito et al. (2015) further noted that blanching treatments improve color by deactivating enzymes, leaching out sugars and amino acids, and enhancing color with salt soaking before frying. Conversely, untreated varieties like *Jalane, Dagim, Gudane, Bubu*, and *Belete* received low color ratings due to browning reactions, such as enzymatic oxidation, caramelization, and the Maillard reaction, as reported by Mosneaguta (2012), Pedreschi (2012), and Wil van Loon (2012).

**TABLE 4:** Score values for sensory characteristics of potato French fries.

Factors		Sensory properties			
Pretreatments	Varieties	Color	Flavor	Crispness	Overall acceptability
Soaked in 3% of NaCl concentration for 50 min	*Belete*	4.60 ± 0.12^abc^	4.87 ± 0.12^ab^	4.94 ± 0.05^a^	4.75 ± 0.06^a^
	*Burka*	5.00 ± 0.14^a^	4.87 ± 0.12^ab^	4.93 ± 0.06^a^	4.71 ± 0.01^a^
	*Bubu*	4.35 ± 0.05^bcd^	4.73 ± 0.23^abc^	4.71 ± 0.01^ab^	4.51 ± 0.07^bcd^
	*Dagim*	4.15 ± 0.06^bcd^	4.60 ± 0.35^abc^	4.39 ± 0.01^cd^	4.25 ± 0.05^efg^
	*Gudane*	4.33 ± 0.06^bcd^	4.73 ± 0.23^abc^	4.71 ± 0.01^ab^	4.44 ± 0.07^cde^
	*Jalane*	3.79 ± 0.19^d^	4.53 ± 0.40^abc^	4.13 ± 0.06^def^	4.05 ± 0.12^g^
Blanched in hot water at 85°C ± 2°C for 5 min	*Belete*	4.71 ± 0.15^ab^	4.93 ± 0.06^a^	4.76 ± 0.05^ab^	4.80 ± 0.06^a^
	*Burka*	5.00 ± 0.06^a^	4.93 ± 0.06^a^	4.75 ± 0.05^ab^	4.81 ± 0.01^a^
	*Bubu*	4.56 ± 0.08^abc^	4.80 ± 0.17^ab^	4.51 ± 0.01^bc^	4.62 ± 0.10^abc^
	*Dagim*	4.23 ± 0.20^bcd^	4.66 ± 0.29^abc^	4.23 ± 0.02^cde^	4.33 ± 0.12^def^
	*Gudane*	4.53 ± 0.03^bcd^	4.87 ± 0.12^ab^	4.51 ± 0.01^bc^	4.53 ± 0.02^abcd^
	*Jalane*	4.11 ± 0.13^cd^	4.60 ± 0.35^abc^	4.03 ± 0.02^ef^	4.19 ± 0.11^fg^
Untreated sample (control)	*Belete*	2.44 ± 0.21^ef^	4.63 ± 0.12^abc^	3.93 ± 0.06^f^	3.61 ± 0.06^h^
	*Burka*	3.00 ± 0.20^e^	4.57 ± 0.06^abc^	3.90 ± 0.17^f^	3.57 ± 0.04^h^
	*Bubu*	2.38 ± 0.13^f^	4.50 ± 0.09^abc^	3.10 ± 0.10^g^	3.55 ± 0.04^h^
	*Dagim*	1.94 ± 0.38^fg^	4.27 ± 0.06^bc^	2.39 ± 0.20^h^	2.97 ± 0.05^i^
	*Gudane*	2.32 ± 0.12^fg^	4.47 ± 0.03^abc^	2.93 ± 0.07^g^	3.52 ± 0.03^h^
	*Jalane*	1.78 ± 0.46^fg^	4.13 ± 0.12^c^	2.27 ± 0.23^h^	2.96 ± 0.11^i^

*Note:* Results are mean values of triplicate determination, and means within a column not sharing the same letter are significantly different (*p* < 0.05).

Abbreviation: ±, standard errors.

#### 3.5.2 Flavor

Table 4 indicates, the flavor rating scores (taste and aroma) of the French fries ranged from 4.13 to 4.93, indicating they were generally liked or rated as good. Flavor analysis of fries showed that all products—soaked, blanched, and untreated—were liked by panelists, likely due to the formation of aromatic volatile compounds from the interaction of cooking oils with product components during frying. This aligns with Wil van Loon's (2012) findings, which suggest that strong flavors in fried foods result from the Maillard reaction between amino groups and reducing sugars, creating flavorful volatiles.

#### 3.5.3 Crispness

The organoleptic test scores for crispness ranged from 2.27 for the control sample of the *Jalane* variety to 4.94 for the *Belete* variety treated with 3% salt concentration. According to the Table 4, panelists rated the texture of fries made from *Belete* and *Burka* varieties treated with NaCl solution highest (4.93–4.94/liked), indicating a preference for the crispiness achieved through this treatment. Studies by Arya et al. (2017) and Das et al. (2021) similarly found that immersion in sodium and calcium chloride produces an ideal texture compared to other methods, like blanching. In contrast, control products received the lowest texture ratings due to excessive moisture loss, caramelization, and Maillard reactions that led to an undesirable texture (Zhang 2017).

#### 3.5.4 Overall Acceptability

In this sensory test, panelists rated *Burka* and *Belete* cultivars as the most acceptable overall, with both blanching and soaking pretreatments preferred for enhancing overall appeal.

### 3.6 Correlation Between Oil Uptake and Proximate Composition

According to Table 5, oil uptake during frying exhibited a strong negative correlation with moisture content (*r* = −0.882, *p* ≤ 0.001), suggesting that as moisture is lost due to heat application, oil tends to replace the evaporated water within the food matrix. This process occurs as water escapes in the form of steam, leaving behind porous structures that are subsequently filled with oil through capillary action (Bouchon 2009). Furthermore, oil uptake showed a perfect positive correlation with crude fat content (*r* = 1.000) and a very strong positive correlation with energy content (*r* = 0.991), indicating that increased oil absorption directly contributes to higher fat and caloric levels in the final product.

**TABLE 5:** Correlation between oil uptake and proximate composition.

Parameters	MC	CFC	Energy	UCC	CPC	CAC	CFbC
CFC	−0.893						
Energy	−0.947	0.892					
UCC	0.466	−0.589	−0.506				
CPC	0.111	−0.300	−0.317	−0.422			
CAC	0.269	−0.435	−0.467	−0.320	0.890		
CFbC	−0.420	0.411	0.354	−0.843	0.551	0.540	
Oil uptake	−0.882**	1.000	0.991	−0.592	−0.295	−0.435	0.411

*Note:* Correlation is significant at *p* ≤ 0.05; **correlation is highly significant at *p* ≤ 0.001.

Abbreviations: CAC, crude ash content; CFbC, crude fiber content; CFC, crude fat content; CPC, crude protein content; MC, moisture content; UCC, utilizable carbohydrate content.

## 4 Conclusion

This study evaluated the effects of different potato varieties and pretreatment methods on the physicochemical and sensory properties of French fries. Among the six potato varieties tested—*Belete, Burka, Bubu, Gudane, Dagim*, and *Jalane*—the *Belete* and *Burka* varieties emerged as the most suitable for producing high‐quality French fries. These varieties demonstrated lower oil uptake when subjected to pretreatments, particularly soaking in a 3% sodium chloride solution for 50 min and blanching in hot water at 85°C ± 2°C for 5 min. The pretreatment methods significantly influenced the nutritional and sensory qualities of the fries. Soaking in salt solution not only reduced oil absorption but also enhanced the crispness and overall acceptability of the fries, making them more appealing to consumers. Blanching further improved the color and texture, contributing to a more desirable product. In summary, for large‐scale production of French fries with optimal quality, it is recommended to utilize *Belete* and *Burka* varieties, employing soaking in a 3% NaCl solution followed by blanching. This combination maximizes nutritional value while minimizing oil content, aligning with consumer preferences for healthier fried products.

## Author Contributions

**Tolina Kebede Regasa:** conceptualization (equal); formal analysis (equal); investigation (equal); methodology (equal); resources; validation (equal); visualization (equal); writing – original draft (equal); writing – review and editing (equal). **Tilahun A. Teka:** conceptualization (equal); formal analysis (equal); investigation (equal); methodology (equal); validation (equal); visualization (equal); writing – original draft (equal); writing – review and editing (equal). **Addisalem Hailu Taye:** conceptualization (equal); formal analysis (equal); investigation (equal); methodology (equal); validation (equal); visualization (equal); writing – original draft (equal); writing – review and editing (equal).

## Conflicts of Interest

The authors declare no conflicts of interest.

## Data Availability

The data that support the findings of this study are available from the corresponding author upon reasonable request.
